# Malonyl-proteome profiles of *Staphylococcus aureus* reveal lysine malonylation modification in enzymes involved in energy metabolism

**DOI:** 10.1186/s12953-020-00169-1

**Published:** 2021-01-12

**Authors:** Yanan Shi, Jingjing Zhu, Yan Xu, Xiaozhao Tang, Zushun Yang, Aixiang Huang

**Affiliations:** 1 College of Food Science and Technology, Yunnan Agricultural University, Kunming, 650201 Yunnan China; 2 Yunnan Center for Disease Control and Prevention, Kunming, 650201 Yunnan China

**Keywords:** Post-translational modification (PTM), Lysine malonylation, *Staphylococcus aureus*, Energy metabolism, Enzymes

## Abstract

**Background:**

Protein lysine malonylation, a novel post-translational modification (PTM), has been recently linked with energy metabolism in bacteria. *Staphylococcus aureus* is the third most important foodborne pathogen worldwide. Nonetheless, substrates and biological roles of malonylation are still poorly understood in this pathogen.

**Results:**

Using anti-malonyl-lysine antibody enrichment and high-resolution LC-MS/MS analysis, 440 lysine-malonylated sites were identified in 281 proteins of *S. aureus* strain. The frequency of valine in position − 1 and alanine at + 2 and + 4 positions was high. KEGG pathway analysis showed that six categories were highly enriched, including ribosome, glycolysis/gluconeogenesis, pentose phosphate pathway (PPP), tricarboxylic acid cycle (TCA), valine, leucine, isoleucine degradation, and aminoacyl-tRNA biosynthesis. In total, 31 malonylated sites in *S. aureus* shared homology with lysine-malonylated sites previously identified in *E. coli,* indicating malonylated proteins are highly conserved among bacteria. Key rate-limiting enzymes in central carbon metabolic pathways were also found to be malonylated in *S. aureus*, namely pyruvate kinase (PYK), 6-phosphofructokinase, phosphoglycerate kinase, dihydrolipoyl dehydrogenase, and F1F0-ATP synthase. Notably, malonylation sites were found at or near protein active sites, including KH domain protein, thioredoxin, alanine dehydrogenase (ALD), dihydrolipoyl dehydrogenase (LpdA), pyruvate oxidase CidC, and catabolite control protein A (CcpA), thus suggesting that lysine malonylation may affect the activity of such enzymes.

**Conclusions:**

Data presented herein expand the current knowledge on lysine malonylation in prokaryotes and indicate the potential roles of protein malonylation in bacterial physiology and metabolism.

**Supplementary Information:**

The online version contains supplementary material available at 10.1186/s12953-020-00169-1.

## Background

The emerging advancements in high-sensitive mass spectrometry and high-quality pan-anti-acyl-lysine antibody have revealed post-translational modifications (PTMs) in the bacterial gene expression, virulence, and protein functions [1, 2]. So far, several PTM types have been identified, i.e., acetylation, crotonylation, succinylation, glutarylation, and 2-hydroxyisobutyrylation. Protein acetylation, a highly-conserved PTM, targets enzymes involved in central carbon metabolism in eukaryotes and various prokaryotes [3, 4]. Moreover, protein acetylation contributes to secondary metabolism, fatty acid metabolism, protein localization regulation, and synthesis in *Thermus thermophilus* [5], *Salmonella enterica*, and certain Gram-positive bacteria, such as *Bacillus subtilis* and *Geobacillus kaustophilus* [6, 7]. Zhang et al. first identified 14 succinylated proteins with 69 succinylation sites in *Escherichia coli* [8]. Subsequently, through the comprehensive analysis of lysine succinylomes in bacteria, succinylation has been characterized to occur in glycolysis, tricarboxylic acid (TCA) cycle, and fatty acid metabolism [9]. Recent studies have described a novel post-translational modification, namely lysine-2-hydroxyisobutyrylation (Khib), on histones of eukaryotic cells with potential involvement in cell transcription and metabolism. Dong et al. found that a lysine de-2-hydroxyisobutyrylase (CobB) is involved in glycolysis regulation by regulating enolase catalytic activities, which further affects bacterial growth [10].

Malonylation, a newly described PTM by means of pan anti-malonylated lysine antibodies and synthetic peptides, involves malonyl-CoA as a cofactor and allows cells to respond to internal and external cues rapidly. Malonylated lysine is negatively charged due to the presence of a carboxylic group, which can impact protein function and regulatory enzymes [11]. Protein acylation modification has been shown to participate in the regulation of enzymatic activity, gene expression, virulence, protein synthesis, translation, and stability, as well as in other biochemical processes. Although malonyl-CoA is considered the most common donor of the malonyl group, malonylation mediating enzymes are still unexplored [11]. SIRT5, a member of the family of lysine deacetylases (KDACs), was found to catalyze lysine de-malonylation reaction in mammalian cells [12]. Therefore, it is speculated that both protein acetylation and malonylation are reversibly regulated by lysine acetyltransferases (KATs) and KDACs in mammalian cells. The addition of acetyltransferase inhibitors or histone deacetylases in vitro can significantly inhibit the growth of *Trichophyton rubrum* by inducing cell apoptosis, or it may point towards a new direction to treat fungal diseases. There has been an increasing interest in exploring the regulatory roles of lysine malonylation (Kmal) in several microbial species, such as *Escherichia coli* [13], *Bacillus amyloliquefaciens* [14], and *Saccharopolyspora erythraea* [15]*.* However, little is known about the substrates and biological roles of Kmal in *S. aureus*.

*Staphylococcus aureus* is a Gram-positive bacterium and the third most common foodborne pathogen worldwide [16, 17]. Previous studies have shown that raw milk and dairy products are heavily contaminated by *S. aureus* [18, 19]. Furthermore, *S. aureus* isolated from several products of animal origin has been shown be to resistant to antibiotics, such as methicillin, vancomycin (glycopeptide), daptomycin (lipopeptide), linezolid (oxazolidinone), tedizolid (anoxazolidinone), dalbavancin (lipoglycopeptide), ceftaroline (β-lactam antibiotic) and carbapenems [20, 21]. Once *S. aureus* contaminates foods, it can multiply under favorable conditions and secrete enterotoxins, which, if ingested, can lead to symptoms of food poisoning, e.g., vomiting. Therefore, *S. aureus* is an important public health concern.

Therefore, the aim of this study was to characterize malonylated residues in proteins of *S. aureus* using affinity enrichment coupled with mass spectrometry-based techniques. To our knowledge, this study is the first to characterize protein lysine malonylation in *S. aureus,* and the present findings provide insights into its biological roles in bacterial energy metabolism.

## Methods

### Bacterial strain and cell culture

*S. aureus* DC.RB_015 was obtained from the Yunnan Center for Disease Control and Prevention, China, and was used throughout the study. *S. aureus* was inoculated in 100 mL of Luria Bertani (LB) broth in a five-hundred-mL Erlenmeyer baffled flask under shaking at 150 rpm for 18 h at 37 °C. Cells at stationary phase (OD_600_ = 0.9) were harvested by centrifugation at 8000×*g* for 5 min at 4 °C. Before lysis, cells were washed twice with cold PBS.

### Protein extraction

Harvested *S. aureus* cells were frozen by liquid nitrogen and ground into powder, then transferred to a five-mL centrifuge tube. Pellets were resuspended in lysis buffer—8 M Urea in 50 mM NH_4_HCO_3_, pH 8.0, containing 1% protease inhibitor cocktail; for PTM experiments, deacetylase inhibitors were also added to lysis buffer, e.g., 3 μM trichostatin A (TSA) and 50 mM nicotinamide (NAM)—and sonicated for 5 min. After incubation on ice for 30 min, the lysate was sonicated again to disrupt DNA clump. After centrifugation at 12,000×*g* at 4 °C for 10 min, supernatants were transferred to new tubes, and protein was quantified by BCA protein assay kit (Beyotime Biotechnology, China).

### Western blotting

Extracted proteins were standardized to the same concentration (2.39 mg/mL) and boiled in SDS loading buffer for 10 min. Proteins were then subjected to 12% SDS-PAGE and transferred to a polyvinylidene difluoride (PVDF) membrane. The membrane was blocked for 2 h in TBS buffer (25 mM Tris-HCl, pH 8.0, 150 mM NaCl) containing 5% bovine serum albumin (BSA) with further incubation overnight at 4 °C with the following: anti-succinyl lysine antibody (catalog no. PTM-419, Lot: 105032317G009, Biolabs Inc., Hangzhou, China) (1:1000, in TBS with 2.5% BSA); anti-malonyl lysine antibody (catalog no. PTM-902, Lot: 23056103HA07, Biolabs Inc.) (1:1000, in TBS with 2.5% BSA); anti-acetyl lysine antibody (catalog no. PTM-101, Lot: 10167 J809, Biolabs Inc.) (1:1000, in TBS with 2.5% BSA); and anti-2-hydroxyisobutyryllysine antibody (catalog no. PTM-802, Lot: 13592312JB09, Biolabs Inc.) (1:1000, in TBS with 2.5% BSA). After three consecutive washes with TBST buffer (25 mM Tris-HCl, pH 8.0, 150 mM NaCl, 0.1% Tween20), the membrane was incubated with goat anti-mouse IgG (H + L) antibody horseradish peroxidase conjugate (1:5000; Thermo Fisher Scientific, Waltham, MA, USA) for 1 h at 37 °C. After washing the membrane three times, an ECL substrate kit was used for protein visualization [14].

### Trypsin digestion

Protein samples were reduced with 5 mM dithiothreitol for 30 min at 56 °C and alkylated with 11 mM iodoacetamide for 15 min at room temperature in darkness. Reduced proteins were diluted by the addition of 100 mM TEAB with urea concentration less than 2 M. Trypsin was added at a 1:50 trypsin/protein ratio for first digestion overnight and then at a 1:100 ratio for second four-hour digestion [14]. A total of 12 mg of proteins was used for trypsin digestion.

### HPLC fractionation

Tryptic peptides were fractionated by high pH reversed-phase high performance liquid chromatography (HPLC) as previously described [15] using a BetaSil™ C18 column (5 μm particle size, 10 mm ID, 250 mm length; Thermo Scientific). Peptides were separated with an acetonitrile gradient (2–60%) in 10 mM ammonium bicarbonate (pH 8.5) for 80 min, 0.7%/min. Peptides were then combined into 10 fractions and vacuum-dried for affinity enrichment.

### Affinity enrichment

Fractionated peptides were dissolved in NETN buffer (100 mM NaCl, 1 mM EDTA, 50 mM Tris-HCl, 0.5% NP-40, pH 8.0) and incubated overnight with 10 μL of drained pre-washed antibody beads (Catalog no. PTM-904; lot: TAJB09B02; PTM Biolab, China) at 4 °C under gentle shaking for lysine-malonylated peptide enrichment. Beads were gently washed four times with NETN buffer and twice with double-distilled H_2_O. Peptides bound to the beads were eluted with 0.2% TFA and then vacuum-dried. Eluted peptides were desalted with C18 tips (Millipore, Billerica, MA, USA), according to manufacturer’s instructions [13].

### Liquid chromatography-tandem mass spectrometry analysis

Enriched lysine-malonylated peptides were dissolved in solvent A (0.1% FA in 2% ACN) and loaded onto a homemade reversed-phase precolumn (75 μm ID × 4 cm in length, 5 μm particle size). The gradient increased from 6 to 23% solvent B (0.1% formic acid in 98% acetonitrile) during the initial 26 min: from 23 to 35% during 8 min, climbing to 80% within 3 min, and then keeping at 80% for the last 3 min; all gradient changes were performed at a constant flow rate of 400 nL/min in an EASY-nLC 1000 UPLC system. Peptides were subjected to a NanoSpray Ionization source followed by tandem mass spectrometry (MS/MS) in a Q Exactive™ Plus system (Thermo Scientific) coupled online with ultra-performance liquid chromatography using the following parameters: 2.0 kV electrospray voltage, 350 to 1800 m/z range for a full scan, and intact peptides were detected in Orbitrap (Thermo Scientific) under a 70,000 resolution. Peptides were then selected for MS/MS using normalized collision energy value set at 28, and fragments were detected in Orbitrap at a 17,500 resolution. A data-dependent procedure that alternated between one MS scan followed by 20 MS/MS scans with 15.0 s dynamic exclusion [13].

### Database search

The resulting MS/MS data were processed using the MaxQuant search engine (v.1.5.2.8). Tandem mass spectra were searched against the UniProt *Staphylococcus aureus* taxonomy database concatenated with a reverse decoy database. Trypsin/P was specified as the cleavage enzyme allowing up to four missing cleavages. Mass tolerance for precursor ions was set at 20 ppm in an initial search and 5 ppm in the main search, and mass tolerance for fragment ions was set at 0.02 Da. Carbamidomethylation on cysteine residues was specified as fixed modification, and malonylation and oxidation on methionine residues were specified as variable modifications. False discovery rate (FDR) was adjusted to < 1%, and the minimum score for modified peptides was set > 40, peptides for quantification: Unique & razor peptides.

### Bioinformatic analysis

The subcellular localization of modified proteins was predicted by WoLF PSORT (version PSORT/PSORTII) [22]. Predictions of amino acid positions and protein secondary structures were performed using, respectively, motif-x and NetSurfP software [23]. Similarly, non-malonylated amino acids in protein secondary structures were analyzed with the Swiss-Prot “*Staphylococcus aureus*” dataset as the negative set. Significance level (*P* value) was set at 0.05 or lower [24]. Preference of flanking sequences of Kmal sites was detected using iceLogo (version 1.2). In total, 21 peptides containing Kmal sites in the central lysine site and 10 neighboring amino acid residues on both sides (21 amino acids in total) were selected as positive sets for analysis. Cytoscape (version 3.3.0) software based on the STRING database (version 11.0) was used to analyze protein−protein interactions of identified malonylated proteins. Interactions that showed confidence score higher than or equal to 0.7 from the STRING database were exported to Cytoscape for analysis. Molecular Complex Detection (MCODE) plugin in Cytoscape was used for the analysis of densely connected regions [25]. InterPro (http://www.ebi.ac.uk/interpro/) and InterProScan software were used to annotate functional domains of all identified proteins. KEGG Automatic Annotation Server (KAAS) was used to obtain the KEGG database description. Annotation was mapped against the KEGG pathway database using the KEGG mapper. InterProScan was used to annotate protein gene ontology (GO) based on sequence homology. A two-tailed Fisher’s exact test was applied to GO/KEGG/Domain enrichment analysis of differentially malonylated proteins against all identified proteins. *P* values < 0.05 were considered as statistically significant.

## Results and discussion

### Characterization of the lysine-malonylated proteins in *S. aureus*

**Fig. 1:**
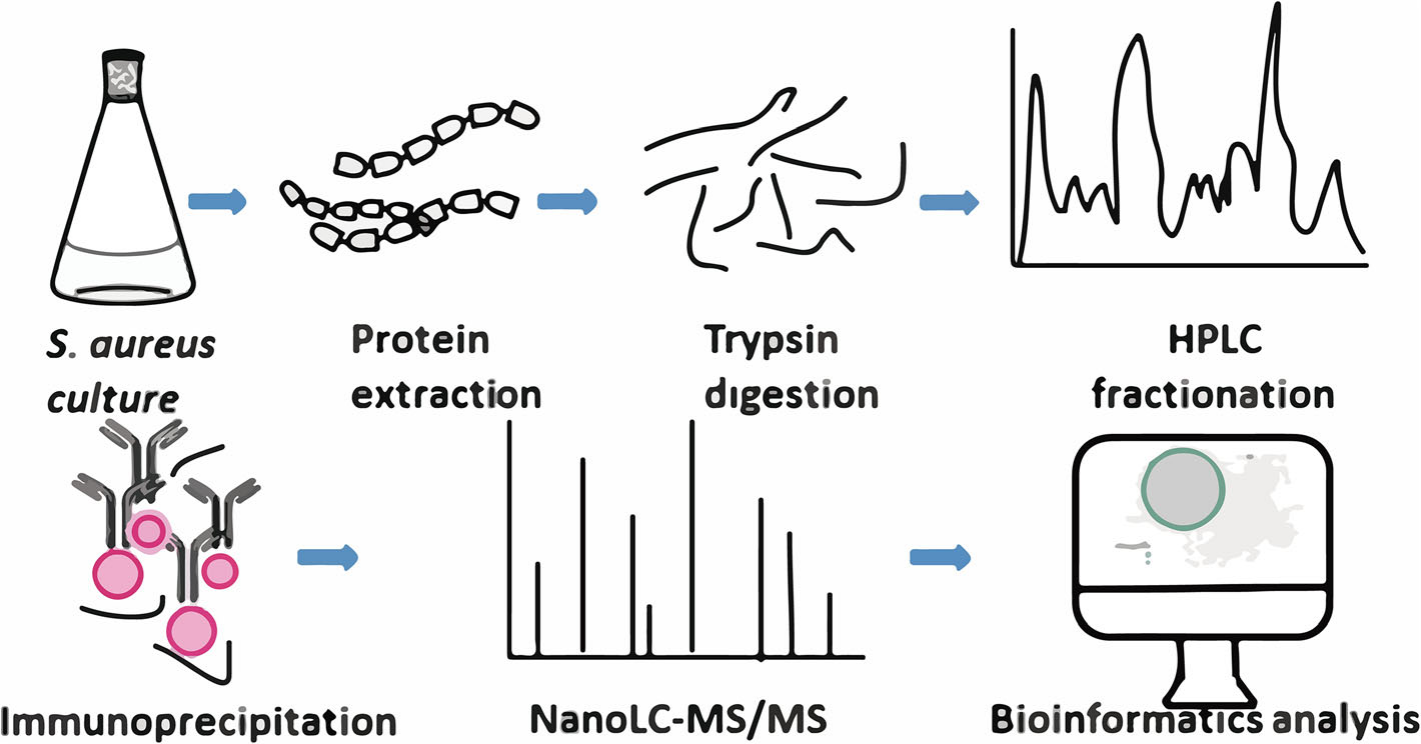
Workflow for the global profiling of lysine malonylation in *S. aureus*

**Fig. 2:**
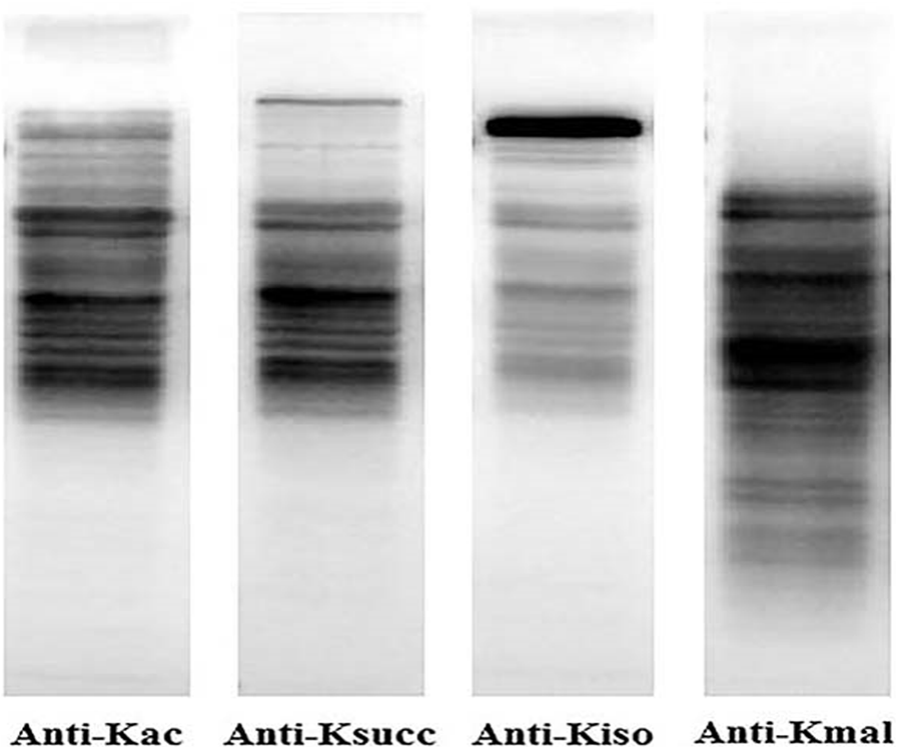
Western blotting analysis of protein lysine malonylation in *Staphylococcus aureus* strain DC.RB_015 with anti-acetyl lysine (anti-Kac), anti-succinyl lysine (anti-Ksucc), anti-2-hydroxyisobutyryl (anti-Kiso), and anti-malonyl lysine antibodies (anti-Kmal)

*S. aureus* cells were collected during the exponential phase, and western blot analysis of whole cell lysates was carried out using pan-anti-acetylation, − 2-hydroxyisobutyrylation, −succinylation, and -malonyl-lysine antibodies. As depicted in Figs. 1 and 2, lysine malonylation is widely spread in the proteome of *S. aureus*. The dot blot analysis was performed to control the specificity of the beads (Fig. S5). Later, global malonylome analysis of *S. aureus* proteome was performed using affinity enrichment followed by high-resolution LC-MS/MS, as previously reported [26] (Fig. S1)

A total of 440 Kmal sites were identified in 281 proteins of *S. aureus* (Supplementary Table S1), which were less than the number of malonylated proteins recently identified in *E. coli* and *B. amyloliquefaciens* [14]*,* but more than in *S. erythraea* [15] and *Toxoplasma gondii* [27]. Among malonylated proteins of *S. aureus*, 191 proteins (67.9%) contained a single Kmal site, 51 proteins (18.1%) contained two Kmal sites, and the remaining had three or more Kmal sites (Fig. S3). Dihydrolipoyl dehydrogenase contained the largest number of malonylated sites (*n* = 7) with a single protein. The second highly malonylated proteins contained six Kmal sites, including phosphoglycerate kinase (PGK) and alanine dehydrogenase (ALD), which are involved, respectively, in the second stage of glycolysis and oxidative deamination of alanine. In the present study, lysine malonylation was found on certain proteins of the 50S ribosomal family (L1, L9, L3, L6, L30), and five Kmal sites were also identified in heat shock protein 70 (Hsp70). Previous studies have shown that heat shock proteins are highly succinylated, containing up to 17 independent lysine residues, and are crucial for host immune response regulation during infection by *Plasmodium falciparum* [28]. The previously reported dihydrolipoyl dehydrogenase had the most intensively acetylated proteins with 15 Kmal sites in *Saccharopolyspora erythraea*.

GO analysis revealed that malonylated proteins of *S. aureus* were shown to be preferably located in the cytosol (54.7%) (Supplementary Fig. S4), or as intracellular non-membrane-bounded organelle (11.3%), ribonucleoprotein complex (14.8%), cytosolic ribosome (9.6%), and ribonucleoprotein complex (9.6%). Several malonylated proteins have been identified in the nucleus, cytoplasm, mitochondria, and chloroplast, thus indicating that a wide variety of biological processes can be potentially regulated by lysine malonylation.

### Malonylated proteins in *S. aureus* are involved in central carbon metabolism

**Fig. 3:**
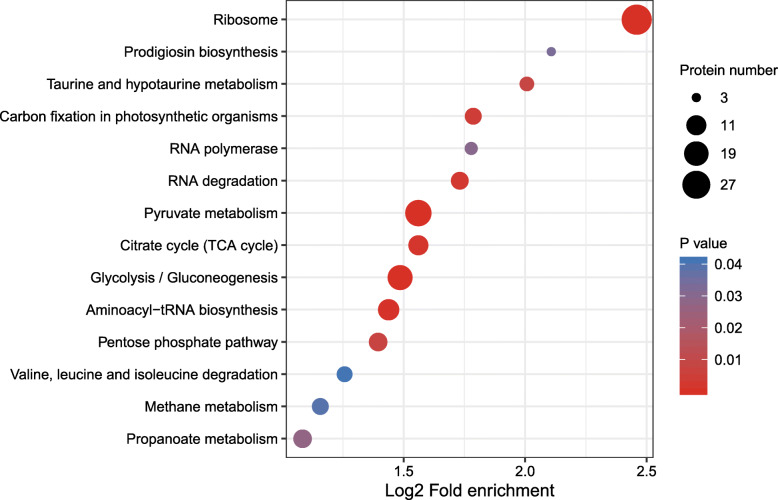
KEGG-based enrichment analysis for lysine-malonylated proteins in *Staphylococcus aureus* strain DC.RB_015

A growing body of evidence suggests that lysine malonylation plays a major role in bacterial metabolism regulation. Malonylome analysis of mammalian cells revealed that SIRT5 regulates both cytosolic and mitochondrial proteins with glycolysis as the targeted pathway. KEGG pathway analysis showed that six categories were highly enriched (Fig. 3): ribosome, glycolysis/gluconeogenesis, pentose phosphate pathway (PPP), tricarboxylic acid cycle (TCA), valine, leucine, isoleucine degradation, and aminoacyl-tRNA biosynthesis. Malonylated proteins related to the ribosome pathway were significantly enriched, suggesting a potential involvement of lysine malonylation in protein synthesis. Enrichment of glycolysis/gluconeogenesis, pyruvate metabolism, and citric acid (TCA) cycle KEGG pathways were also observed in *E. coli*, *S. erythraea*, *Fragaria vesca*, and human cells, suggesting that malonylation may control activity or stability of enzymes involved in those pathways and affect the energy metabolism regulation.

**Fig. 4:**
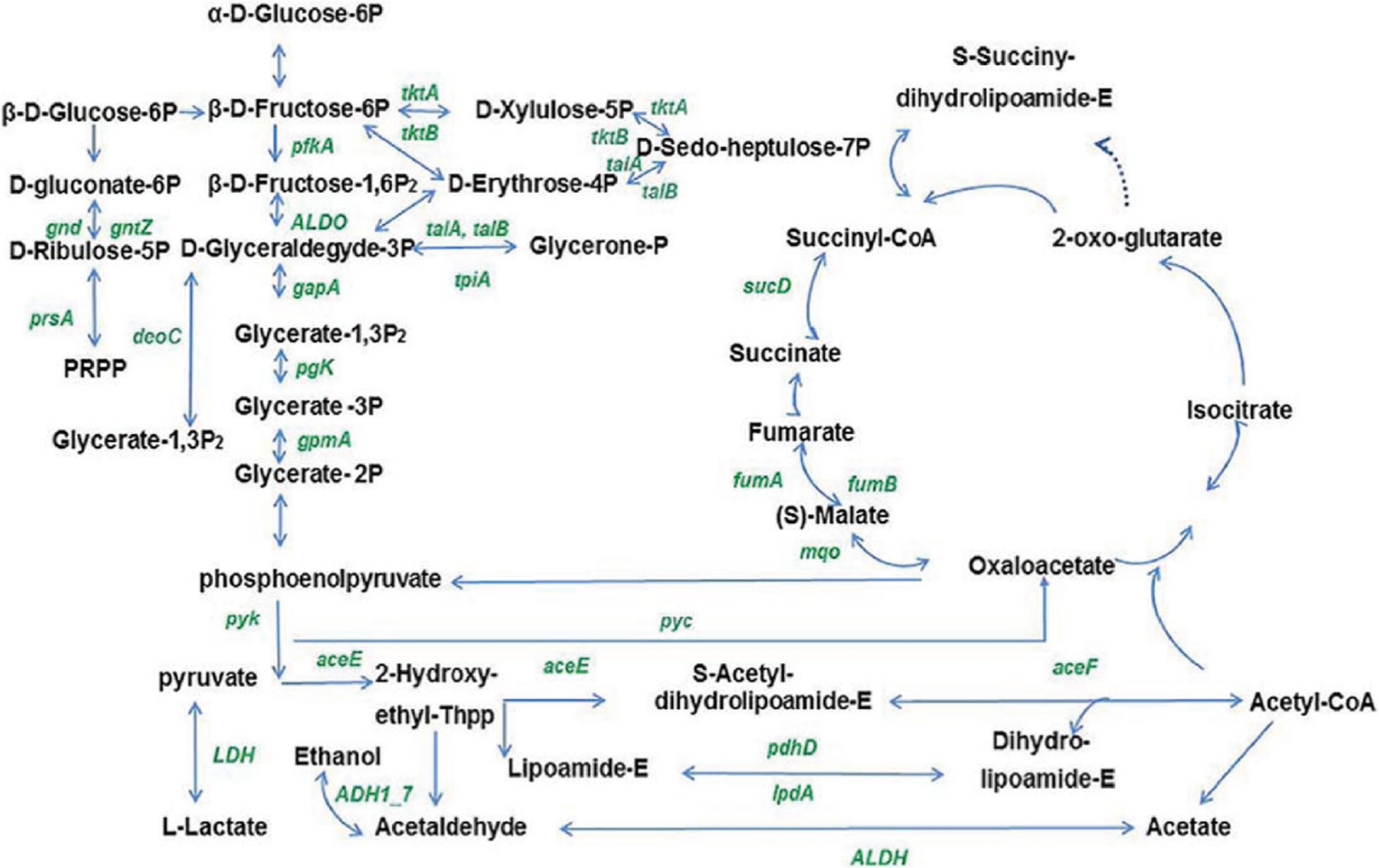
Lysine-malonylated enzymes involved in central carbon metabolism (glycolysis, tricarboxylic acid cycle, pentose phosphate pathway) in *Staphylococcus aureus* strain DC.RB_015. Identified malonylated enzymes are shown in green

Interestingly, nearly all enzymes involved in glycolysis were malonylated at one or more sites (Fig. 4), including critical enzymes 6-phosphofructokinase (PfkA), PGK, and pyruvate kinase (PYK); the latter was found to be malonylated at K66, K433, K434, K435, and K437. Fructose-bisphosphate aldolase plays a central role in glycolysis/gluconeogenesis and may serve as a potential target to fight pathogenic bacteria, and was also found to be malonylated in *S. aureus*. Moreover, the other six malonylated proteins were found to belong to the pentose phosphate pathway. For instance, LpdA, a subunit of pyruvate dehydrogenase, contained 7 Kmal sites, being also highly malonylated in *E. coli* (15 Kaml sites). Additionally, three enzymes involved in the TCA cycle, including succinyl-CoA synthetase, fumarate hydratase, and malate dehydrogenase, were also lysine-malonylated in *S. aureus*. Other malonylated enzymes in *S. aureus* are involved in pyruvate metabolism, namely dihydrolipoyl dehydrogenase (PdhD), LpdA, fructose-bisphosphate aldolase (ALDO) (Fig. 4). Our findings were consistent with previous results in other prokaryotes for which malonylome analysis has been conducted [29]. Considering that the above-mentioned enzymes are mainly related to energy biosynthesis, malonylation is likely to trigger bacterial energy generation.

**Fig. 5:**
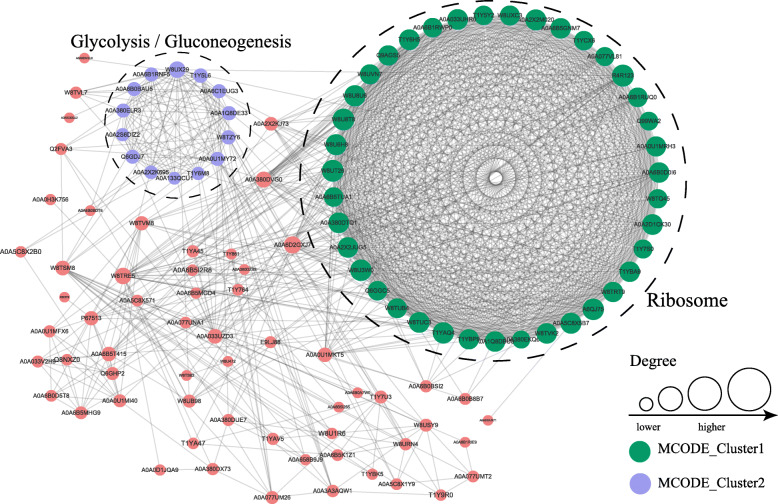
Protein-protein interaction networks of malonylated proteins in *Staphylococcus aureus* strain DC.RB_015. Nodes represent malonylated proteins, and edges represent interactors among malonylated proteins. The color of edges indicates the combined score of interactors

Acyl-lysine modification can regulate protein-protein interaction [15]. PPI analysis in the STRING database and PPI networks visualized in Cytoscape helped identify major biological processes affected by Kmal in *S. aureus* (Fig. 5). A number of highly associated subnetworks of Kmal proteins were revealed, including glycolysis/gluconeogenesis and ribosome-related processes, which is consistent with KEGG pathway enrichment analysis.

### Enrichment analysis of *S. aureus* lysine-malonylated proteins

**Fig. 6:**
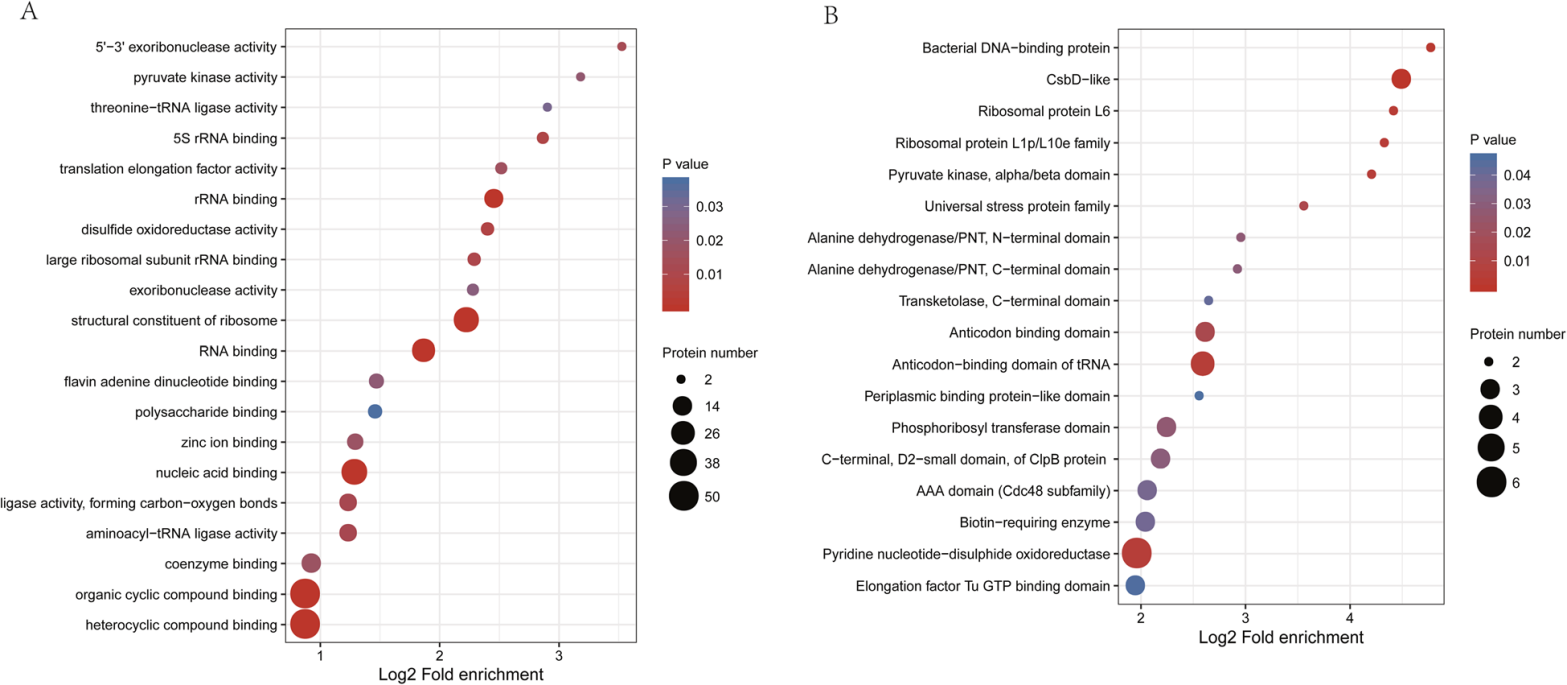
Gene ontology term enrichment and protein domain annotation of malonylated proteins in *Staphylococcus aureus* strain DC.RB_015

Enrichment analysis was performed to determine functional categories for lysine-malonylated proteins in *S. aureus*. Most malonylated proteins in *S. aureus* were significantly related to the following categories: ligase activity, small molecule binding protein, and structural constituent of ribosome (Fig. 6a). Other categories, such as organic cyclic compound binding, heterocyclic compound binding, nucleic acid binding, structural constituent of ribosome, RNA binding, aminoacyl-tRNA synthetases, and elongation factors (FusA, TufA, Efp) were also significantly enriched, suggesting a role of protein malonylation in protein synthesis in *S. aureus*. Additionally, DnaK and Tig, two proteins involved in protein folding and export, respectively, were also malonylated in *S. aureus*. DnaK in *Salmonella Typhimurium* was also found to harbor two Kmal sites (K324, K555) [30]. Synthesized proteins that undergo further modification by chaperones need to find proper localization in the cell by chaperone-mediated transportation. This evidence suggests a potential role of lysine malonylation in controlling protein synthesis in *S. aureus*.

DNA gyrase subunit A (GyrA) in *S. aureus* was found to be malonylated with one modification site. The malonylated protein fructose-bisphosphate aldolase, which plays an essential role in glycolysis and gluconeogenesis pathways, has been considered a potential target for drug development against pathogenic bacteria [31], were malonylated fructose-bisphosphate aldolase containing 2 Kmal sites in K264 and K265. In addition to the regulation of cell metabolism and protein synthesis, lysine malonylation also modulates gene expression.

In this study, RNA polymerase subunits RpoA, RpoB, and RpoC were found to contain Kmal sites. RNA polymerase subunits were also found to be highly acetylated in *E. coli* (15 sites in RpoB, 11 in RpoC, and 2 in RpoA) [32]. Moreover, proteins involved in Rho-dependent termination were also lysine-malonylated in *S. aureus*, suggesting a potential role of lysine malonylation in controlling RNAP promoter specificity, strength, and activity. Additionally, primary free radical scavenging enzymes in *S. aureus*, such as superoxide dismutase, alkylhydroxide peroxidase, thioredoxin, and catalase, also contained malonylated modifications.

The protein domains and functional sites were annotated by InterProScan based on protein sequence alignment using the InterPro database to identify the lysine-malonylated residues in the active protein sites. Among the malonylated peptides in *S. aureus*, the following signatures and active site motifs were found (Supplementary Table S2): 30S ribosomal protein S3 site in KH domain protein; thiol reductase thioredoxin site in thioredoxin; alanine dehydrogenase site in alanine dehydrogenase/PNT, N-terminal domain; pyruvate kinase site in pyruvate kinase alpha/beta domain PYK; pyruvate carboxylase site, biotin carboxyl carrier protein of acetyl-CoA carboxylase site in biotin-requiring enzyme; catabolite control protein site in transcriptional regulator CcpA; pyruvate oxidase site in thiamine diphosphate-dependent CidC; dihydrolipoyl dehydrogenase site in pyridine nucleotide-disulphide oxidoreductase LpdA. Several malonylated peptides were also found as signatures in PnpA (an RNA binding domain profile), GlyS (Anticodon binding domain profile), ALD (alanine dehydrogenase/PNT profile), RplA (ribosomal protein L1p/L10e family) and TufA (elongation factor Tu domain) (Fig. 6b). Those findings pinpoint the preferred location of malonyl groups in *S. aureus* proteins and suggest possible specialized functions of malonylation.

### Pattern analysis of malonylated peptides

**Fig. 7:**
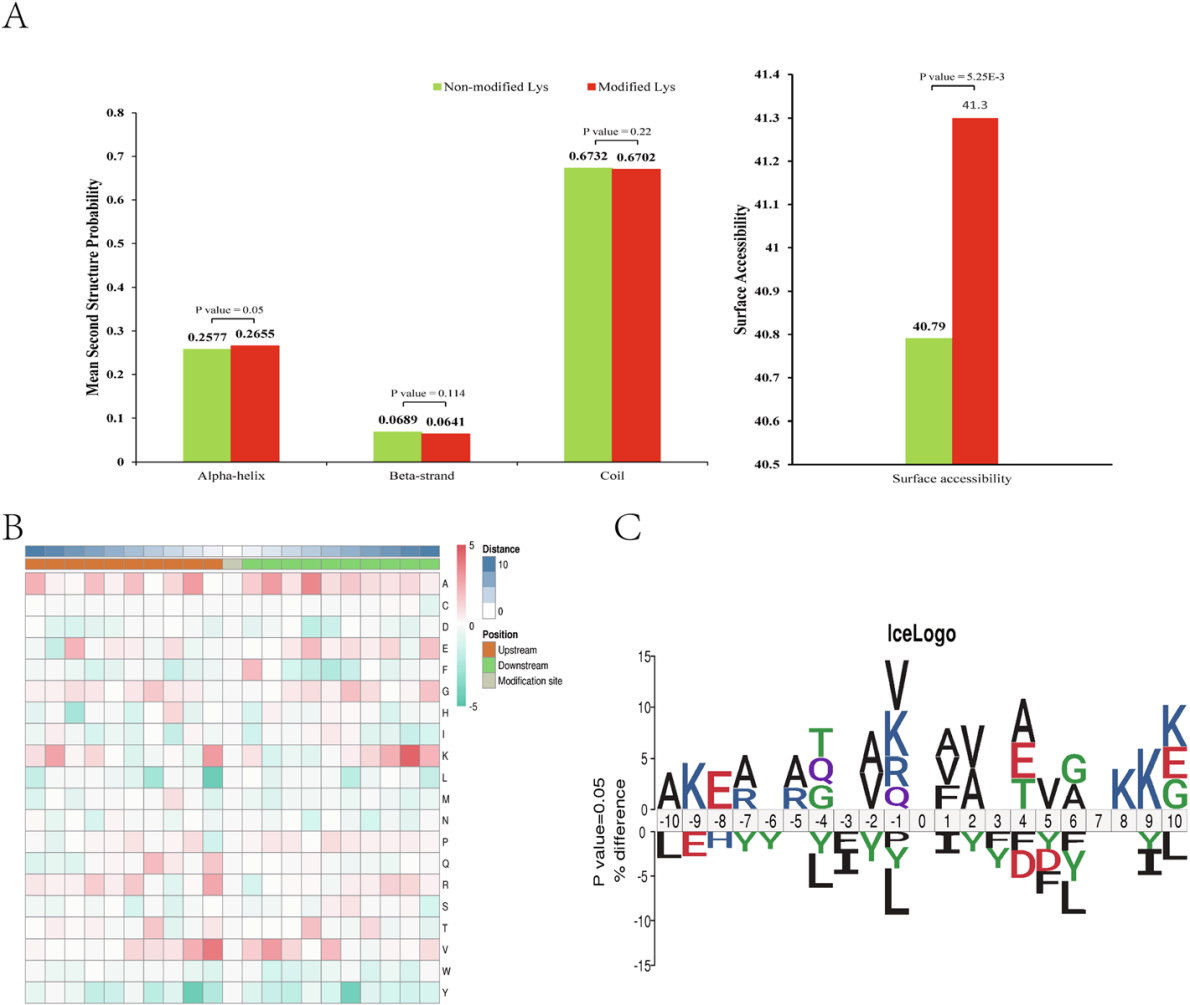
(A) Occurrence of lysine malonylation in protein secondary structures of *Staphylococcus aureus* strain DC.RB_015: alpha-helix, beta-strand, and coli. Predicted surface accessibility of malonylation sites. (B) Heat map of amino acids at positions ranging from − 10 to + 10 around lysine-malonylated residues in *S. aureus*. Red and green colors indicate high and low frequency, respectively. (C) Sequence analysis of the flanking region of lysine-malonylated sites in *S. aureus*

Structural analysis of malonylated peptides in *S. aureus* enabled the identification of surface accessibility of Kmal sites. The average surface accessibility of malonylated lysines was significantly higher (*p* < 0.005) than non-malonylated lysines (Fig. 7a), which also indicates that Kmal sites are preferably located on the surface of proteins. Certain binding proteins may increase the hydrophobicity of large complexes, thereby interfering with normal assembly in an aqueous environment. Lysine malonylation may thus balance the hydrophobicity by enhancing accessibility to the protein surface. Interestingly, 32.96% of Kmal sites were located in secondary structure regions; of them, 26.55% were located in α-helices and 6.41% in β-sheets regions. The remaining 67.02% of malonylation sites in *S. aureus* proteins were located in the unstructured coil regions (Fig. 7a), suggesting that malonylation is more likely to occur in disordered rather than ordered regions in proteins.

Accumulating studies on prokaryotes have proved the preferences for amino-acid residues at particular positions surrounding the acetylated lysine and succinylated lysine [33]. Malonyl-proteins regulated by the same type of enzymes often exhibit similar sequences. Therefore, amino acids around the malonylated lysine from − 10 to + 10 were mapped to determine the specific amino acids adjacent to malonylated lysines. As shown in Fig. 7c, the frequency of valine (V) in position − 1 and alanine (A) at + 2 and + 4 positions was high, which is in agreement with heat map analysis (Fig. 7b). Alanine (A) was overly represented around the malonylated lysine in *S. aureus*, a pattern that is similar to Kmal found in *E. coli* [13].

### Conserved lysine-malonylated proteins in *S. aureus* and *E. coli*

**Fig. 8:**
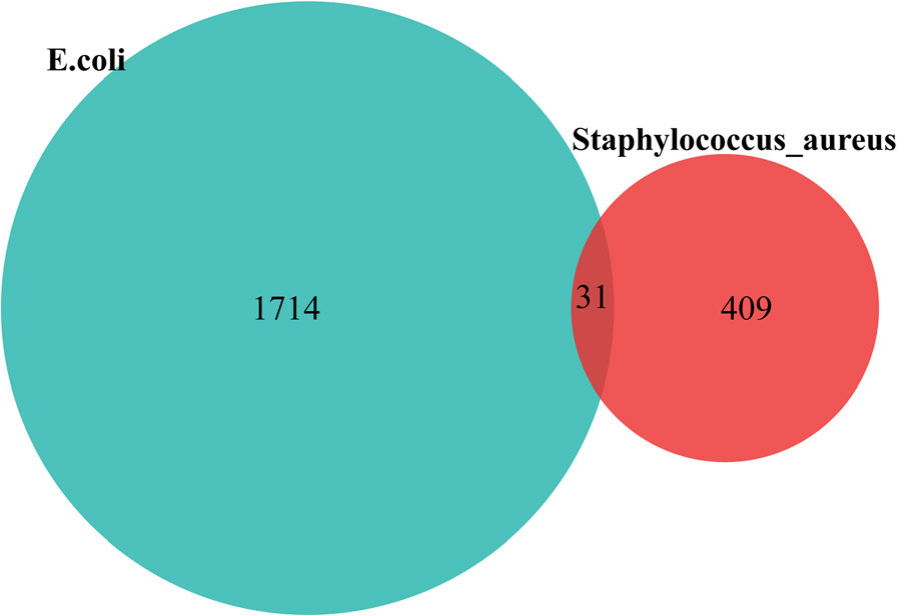
Venn diagram of identified malonylated sites in previously reported *E. coli* malonylomes

Lysine malonylome of *S. aureus* was compared to previously reported *E. coli* malonylomes. Functional classification of *S. aureus* lysine-malonylated proteins is very similar to that of *E. coli* [13]. Interestingly, 31 malonylated sites in *S. aureus* were considered homologous with *E. coli* malonylome (Fig. 8; Supplementary Table S3). Most of the acetylated proteins involved in protein synthesis and energy metabolism in *S. aureus* were considered orthologous to *E. coli.* A small subset of lysine-malonylated proteins was commonly found in these two bacterial malonylomes: DNA-binding protein, glyceraldehyde-3-phosphate dehydrogenase, phosphoglycerate kinase, NADP-dependent phosphogluconate dehydrogenase, ATP synthase, 50S ribosomal protein L1, 50S ribosomal protein L3, glyceraldehyde-3-phosphate dehydrogenase, 30S ribosomal protein S3, dihydrolipoyl dehydrogenase, pyruvate kinase, and formate C-acetyltransferase.

## Conclusions

In the present study, 440 lysine-malonylated sites were described in 281 proteins of *S. aureus*, which confirms the widespread occurrence of lysine malonylation in eubacteria and indicates a conserved PTM shared by eukaryotic and bacterial cells. The findings presented herein expanded the current understanding of lysine malonylation in prokaryotes. More specifically, the malonylated sites were described to occur in key metabolic enzymes involved in several important cellular pathways in *S. aureus*, such as glycolysis/gluconeogenesis, pyruvate metabolism, TCA cycle, and protein synthesis. Moreover, malonylated sites were found at or near active sites of several proteins, indicating the important role of Kmal in the functional regulation of essential proteins in *S. aureus*. Finally, this study provides useful resources for further functional investigations of lysine malonylation in bacteria.

## Supplementary Information

**Additional file 1: Fig. S1.** Identification of malonylated peptides by mass tolerance distribution.

**Additional file 2: Fig. S2.** Identification of malonylated peptides by length.

**Additional file 3: Fig. S3.** Distribution of malonylated proteins based on the number of malonylated peptides.

**Additional file 4: Fig. S4.** Cellular component ontology of identified malonylated proteins.

**Additional file 5: Fig. S5.** Dot blot experiment.

**Additional file 6: Table S1.** Annotated MS/MS spectra of malonylated peptides and sites in *Staphylococcus aureus.*

**Additional file 7: Table S2.** Domain enrichment analysis of proteins corresponding to modification sites in *Staphylococcus aureus*.

**Additional file 8: Table S3.** Conservative analysis of the identified malonylated sites in *Staphylococcus aureus* and previously reported *E. coli* malonylomes.

**Additional file 9 Table S4.** Standardization of protein concentration.

## Data Availability

The datasets used and analyzed during the current study are available from the corresponding authors on reasonable request.
