# Supplementary figures and images for: Malonyl-proteome profiles of Staphylococcus aureus reveal lysine malonylation modification in enzymes involved in energy metabolism
Source: Proteome Sci. 2021 Jan 12;19:1. doi: 10.1186/s12953-020-00169-1 (PMC7802289; doi:10.1186/s12953-020-00169-1)

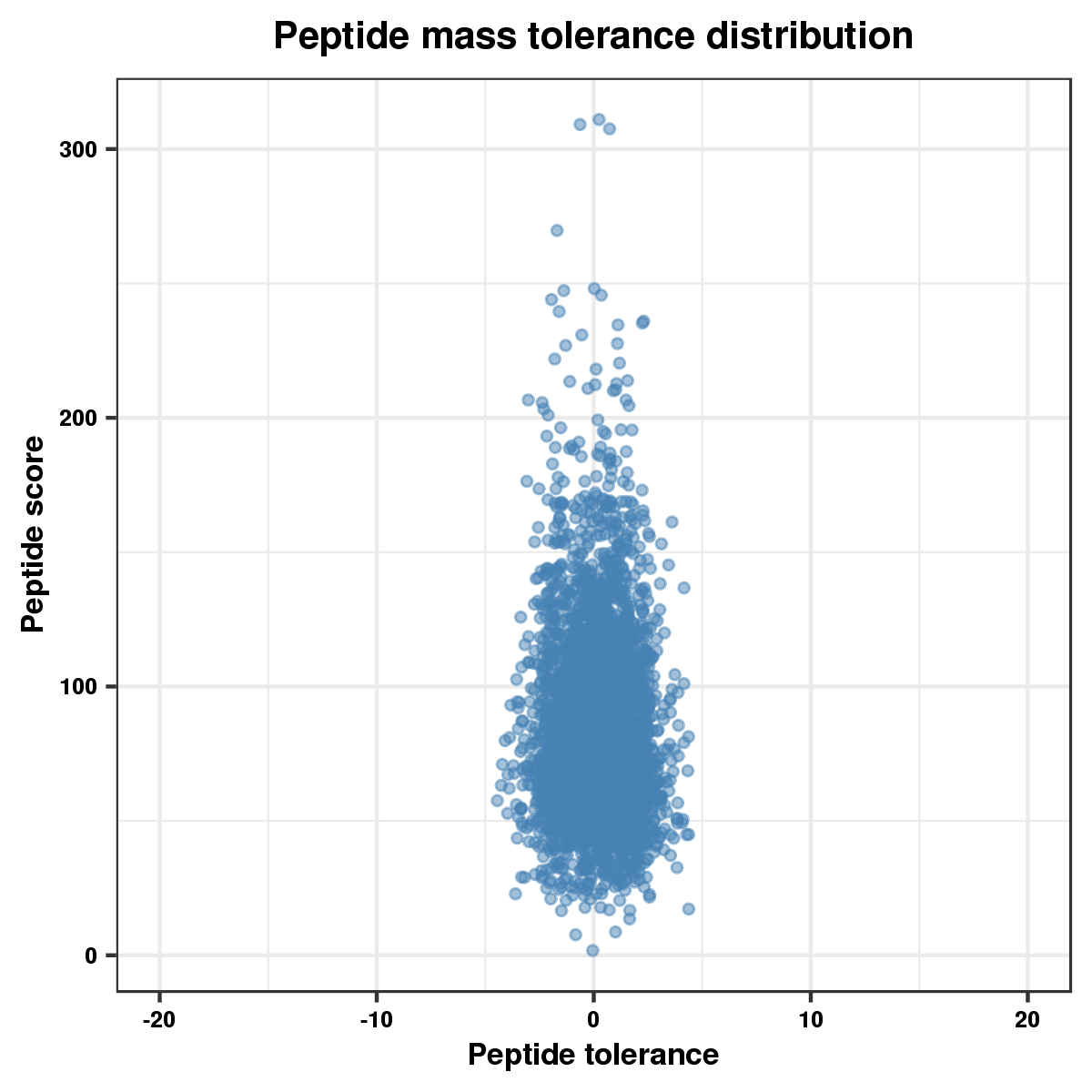

Supplement: Supplementary file 1 — Additional file 1: Fig. S1. Identification of malonylated peptides by mass tolerance distribution. [file 12953_2020_169_MOESM1_ESM.png]

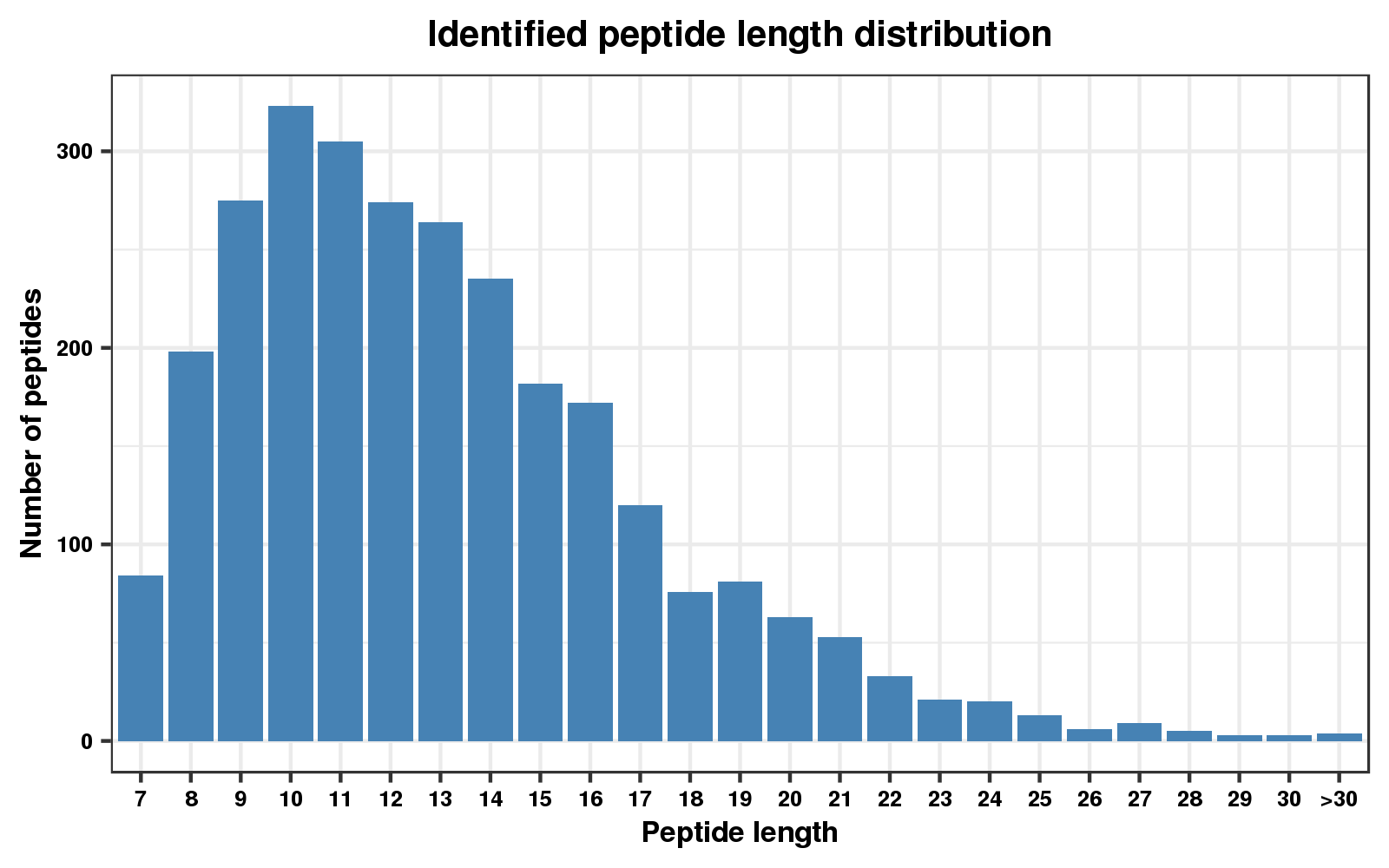

Supplement: Supplementary file 2 — Additional file 2: Fig. S2. Identification of malonylated peptides by length. [file 12953_2020_169_MOESM2_ESM.png]

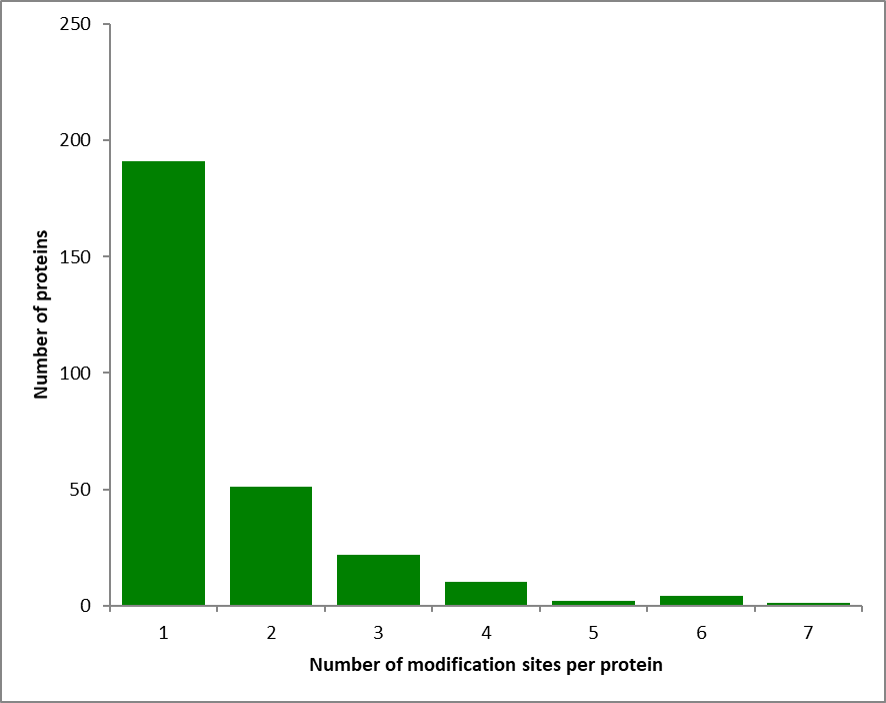

Supplement: Supplementary file 3 — Additional file 3: Fig. S3. Distribution of malonylated proteins based on the number of malonylated peptides. [file 12953_2020_169_MOESM3_ESM.png]

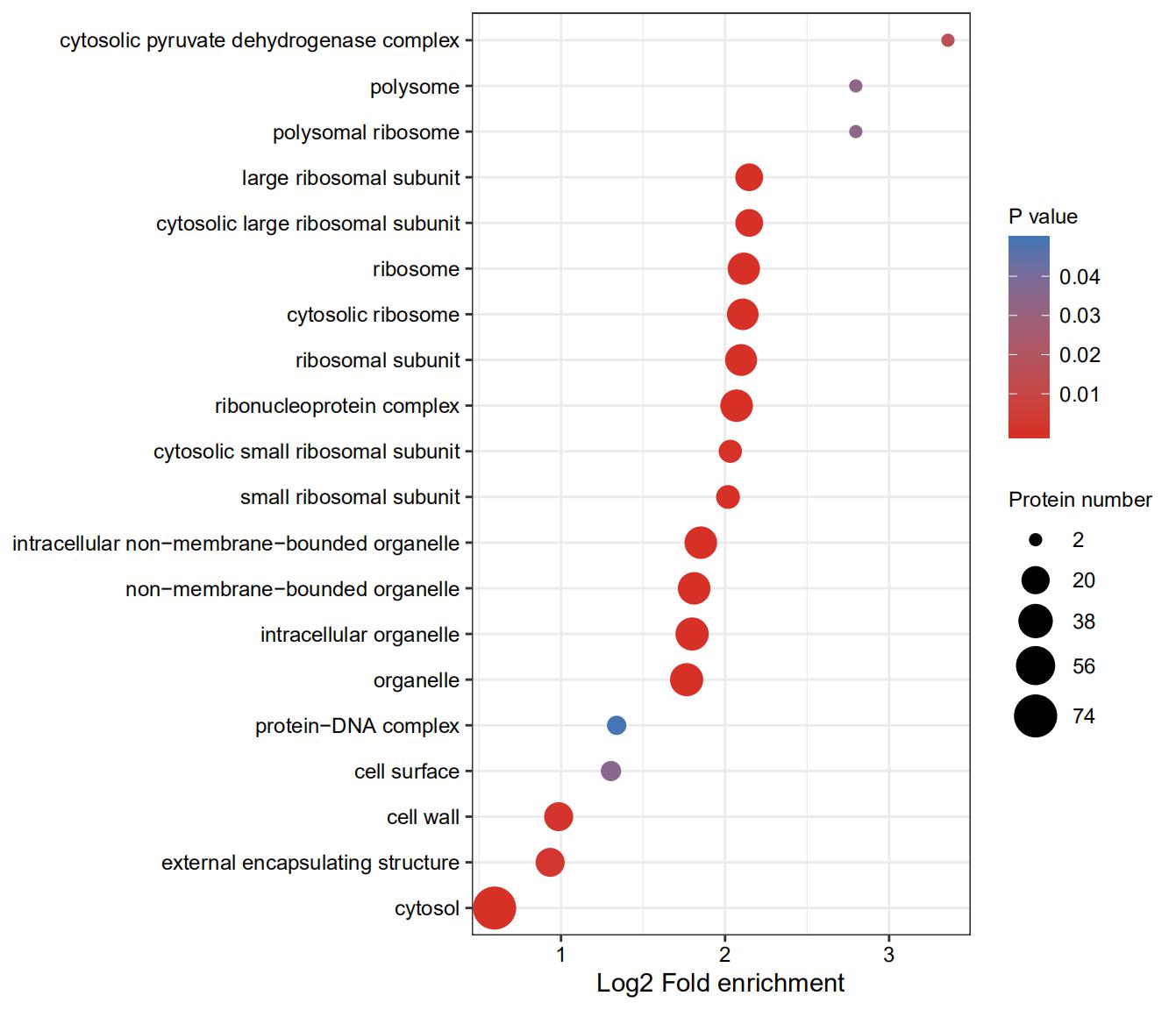

Supplement: Supplementary file 4 — Additional file 4: Fig. S4. Cellular component ontology of identified malonylated proteins. [file 12953_2020_169_MOESM4_ESM.jpg]

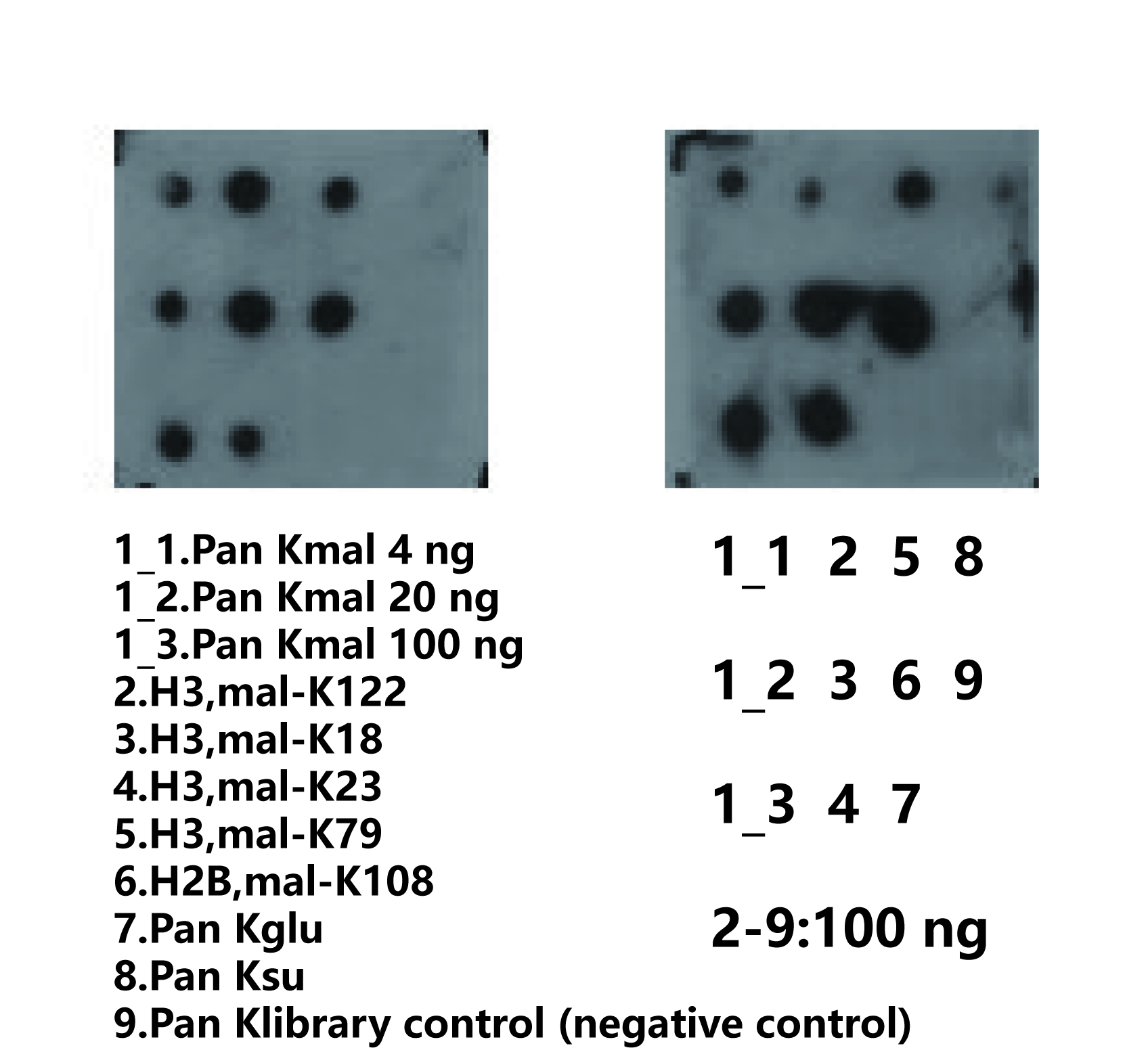

Supplement: Supplementary file 5 — Additional file 5: Fig. S5. Dot blot experiment. [file 12953_2020_169_MOESM5_ESM.tif]
